# Giant intraoral condyloma accuminatum lesion coexisting with genital condyloma

**DOI:** 10.12669/pjms.305.5322

**Published:** 2014

**Authors:** S.Serap Moroglu Ozdamar, Ozkan Ozdamar, Zafer Kucukodaci

**Affiliations:** 1 S.Serap Moroglu Ozdamar, Department of Oral and Maxillofacial Surgery, Faculty of Dentistry, Marmara University, Istanbul, Turkey.; 2 Ozkan Ozdamar, Department of Gynecology and Obstetrics, Golcuk Military Hospital, Kocaeli, Turkey.; 3 Zafer Kucukodaci, Department of Pathology, Gumussuyu Military Hospital, Istanbul, Turkey.

**Keywords:** Condyloma accuminatum, Oral condyloma, Genital condyloma, HPV

## Abstract

The condyloma acuminatum (venereal wart) is a benign epithelial proliferation that occurs most frequently on the mucous membranes of the perianal and genital areas of men and women. The transmissible etiological agents of this lesion are papillomaviruses. In some rare instances these lesion can also be found in the oral cavity. In this 50-year-old woman; a wide rugose, cauliflower-like, exophytic lesion on the attached gingiva in the anterior region thought to be epulis fissuratum or a giant-cell granuloma turned out to be condyloma acuminatum following the excisional biopsy. This patient also had some genital lesions and tested positive for the human papilloma virus which is to be expected due to fact that intraoral presentation of condyloma acuminatum is a lot more frequent in patients who have anogenital lesions according to the literature. This report describes the etiology, diagnosis, treatment and follow-up an intraoral condyloma.

## INTRODUCTION

Condyloma acuminatum, also known as venereal wart, is a relatively common, benign, epithelial proliferation that most commonly located on the mucous membranes of varying tissues with warm, moist squamous epithelial surface. It has been etiologically related to low-risk human papillomaviruses (HPV), mostly HPV types 6 and 11.^1^^,^^2^

Condyloma lesions, which are commonly considered to represent a sexually transmitted infection, frequently involve anogenital region however they might develop at oral mucosa.^1^^-^^3^ Oral sites affected by condylomaaccuminata are the mucosa of the gingiva, cheeks, lips and hard palate or the site of the contact / traumatic event on non-keratinized tissues. Oral cavity involvement might occur without the accompanying anogenital disease.^3^

The clinical appearance of condyloma acuminatum is variable and ranges from small, sessile lesions to larger pedunculated proliferations with smaller satellite growths. The differential diagnosis should also include verruca vulgaris, multiple squamous papillomas and oral mucosal lesions of Cowden’s (multiple hamartoma) syndrome.^4^

Pathological examinations of the infected tissues reveal some prominent clues. The papillomatous projections making up the verrucoid lesion generally show a parakerototic surface with marked underlying acanthosis.^5^ Koilocytosis of upper level epithelial cells is usually found. The epithelial layer itself is hyperplastic without evidence of dysplastic change and these epithelial changes might be mistaken for malignancy. The underlying stroma is well vascularized and may contain a trace of chronic inflammatory cells.^1^^,^^3^

Here we report a rare case of a giant oral condyloma accuminatum coexisting with genital HPV lesions and discuss its surgical treatment.

## CASE PRESENTATION

A 50-year-old woman presented to outpatient clinic of Oral and Maxillofacial Surgery, Marmara University with the complaint of a growing lesion, which she first noticed 5 months earlier, inside her mouth. She reported no smoking history or alcohol use. In her past history, she had two operations, a myomectomy and a total abdominal hysterectomy, but no chronic illnesses or drug use. In addition, she declared to have an intraoral operation due to an epulis lesion, which at the pathological examination was reported as papillomatous lesion, in the region of maxillary 11-12^th^ teeth.

**Fig.1:**
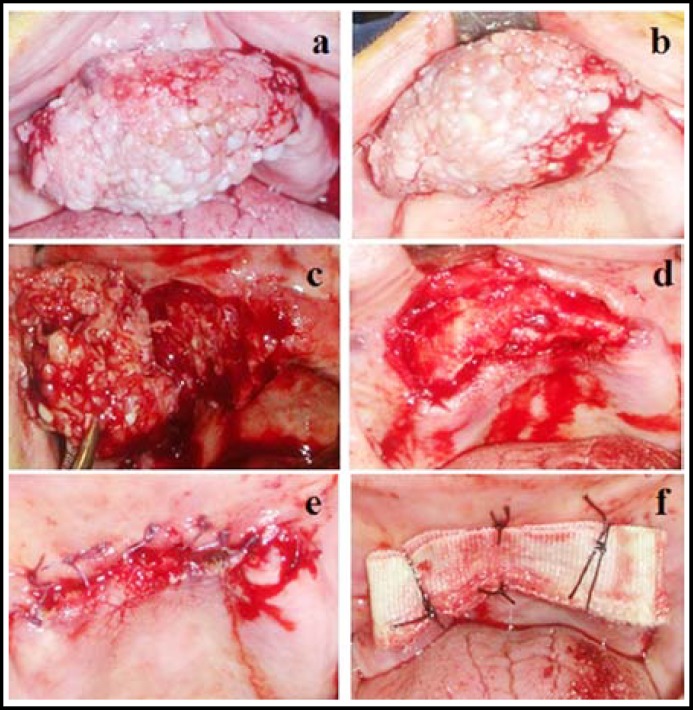
**a, b, **The view of the intraoral lesion located at the hard palate region, **c: **Excision procedure of the lesion, **d,** Postoperative view of the wound site, at the base of the wound bone structure of the hard palate is seen, **e, f, **Early postoperative view after the closure of the wound

Intraoral examination revealed a 2x5x4 cm in size, papillomatous, exophytic, hemorhagic and sessile lesion extending from the maxillary right second premolar region to left upper canine region (Fig. 1a, 1b). As a result of the examination the patient was sent for an operation with preliminary diagnoses of reparative giant cell granuloma, epulis fissuratum and condyloma accuminatum. Queried the presence of any similar lesions elsewhere in the body, the patient also mentioned about some wart–like lesions in her genital area. Thereupon the patient was referred for a gynecological examination which revealed three smooth, exophytic, polypoid, sessile lesions measured approximately 1.5x1.0 cm in the largest dimensions on labia majora. The appearance of the uterine cervix was normal, but yet a cervical Pap smear and material for HPV typing were obtained. The lesions were excised with electro surgery and sent to pathology laboratory.

Since such oral lesions might be suggestive of HIV infection, laboratory diagnostics for HIV and other causes of sexually transmitted diseases were requested. The screening test results were negative, surgical excision was arranged.

**Fig.2:**
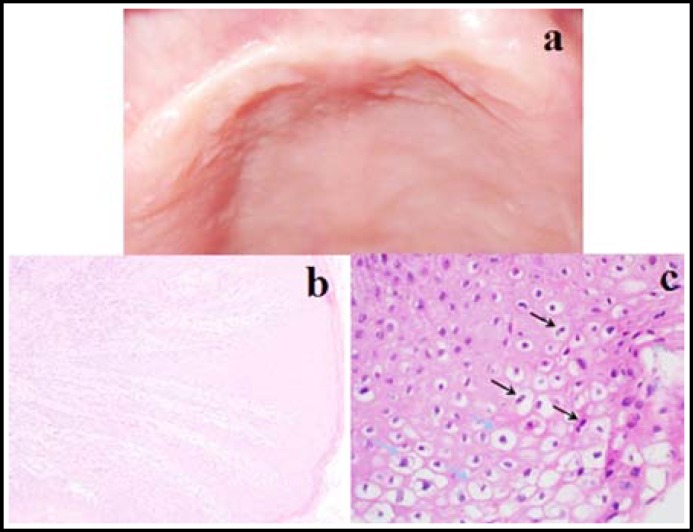
**a,** The appearance of the wound site at postoperative 2^nd^ week, **b,** Photomicrograph of the oral lesion (hematoxylin and eosin stain X100), koilocytic cells are visible in the epithelium. **c,** Photomicrograph of the genital lesion (hematoxylin and eosin stain X400), black arrows show binucleation and blue arrows show perinuclear halo

Following the local anesthesia of the involved intraoral region, the mass was removed with a wide excision (Fig. 1c, 1d). After the hemostasis was established, the wound edges were approximated with 2/0 polyglycolide (Pegelak; Doğsan®, İstanbul) (Fig.1e). A gauze with antibiotic ointment was planted to the wound site that was left for secondary healing (Fig.1f). Placed into a 10% formalin solution, the excised tissue was sent to laboratory for pathological examination. Cervical Pap smear test was negative for any malignant or pre-malignant cervical lesions and HPV typing was positive for HPV DNA type 11 and 32. The patient was periodically controlled once every two days for the first postoperative week and consequentlyon 2^nd^week, 1^st^, 3^rd^ and 6^th^ months (Fig.2a). Pathological examination of the oral lesion confirmed the diagnosis, which revealed condylomaacuminatum (Fig.2b). Genital lesions were confirmed to be of HPV infection (Fig.2c).

After surgical treatment, the patient was informed about possible recurrences which are common and presumably are related to the surrounding appealingly normal tissue that may act as a host for the infectious agent.

## DISCUSSION

HPV infection is the most common and widespread viral infection transmitted by close and repeated sexual contact. Transmission of HPV ordinarily requires sexual contact with the genital skin, mucous membranes or body fluids of a partner with either warts or subclinical infection.^6^ The Centers for Disease Control and Prevention estimates that the risk of a woman acquiring genital HPV by age 50 is greater than 80 percent.^7^ Although HPV infection may be seen in every age groups, sexually active adults aged 15–24 account for approximately one half of new HPV infections each year.^8^ More than 200 HPV types have now been identified and these types are classified as high-risk and low-risk based upon their cervical cancer oncogenicity. High-risk HPV types are frequently associated with premalignant and malignant lesions however low-risk HPV types 6 and 11, which are rarely oncogenic, cause nearly all genital warts and a minority of subclinical HPV infections. HPV type 11 in addition to type 32 was reported as causative agent in our case. HPV contributes to 90% of anal cancers and 40% of vulva, vaginal, and penile cancers.^9^ Nevertheless, the relation between these viruses and the pathogenic cancer in the oral mucosa remains controversial, and there appears to be more impact of HPV with other chemical and physical carcinogens such as tobacco and alcohol. In our case, as can be seen from the figures (Fig. 1a, 1b), patient’s oral hygiene was quite insufficient. Impaired oral hygienic environments too may contribute to the development of oral condyloma.

Surgical excision, which may be cryosurgery, scalpel excision, electrodesiccation, or laser ablation are the most commonly preferred treatment modalities for these lesions. However they are destructive and non-specific. Condyloma lesions in the oral cavity of this size are extremely rare and in the case encountered in literature^10^, the lesion in the lower lip was surgically removed. In our case we removed the intraoral lesion with a wide surgical excision. It must always be noted that primary suturing of an intraoral lesion of this magnitude might completely eliminate the vestibular sulcus depth. However, given that the bone defect present in the anterior region, it is necessary to prevent the formation of labile crest. Thus, following the bleeding in the operation area taken under control by cauterization, it seems to be a more accurate approach to suture the related region only to approximate the wound edges and to close the wound with an iodoform gauze in order to allow secondary healing. It is quite important to take complete control of bleeding in terms of minimizing any risk of hematoma and thereby wound dehiscence or its infection.

In the postoperative period, one of the most important issues to be aware of is oral hygiene. Our patient, due to having low oral hygiene, was called for control once every two days in the postoperative first week and the wound was irrigated with saline solution. For one week postoperatively, the patient was given antibiotics and anti-inflammatory treatments. We consider that in the postoperative period, the amount of the reduction in the vestibular depth is highly successful for a sessile mass of this magnitude. Furthermore, the formation of any labile crest is not available. As a result, in cases where surgical treatment is fulfilled with a proper postoperative care, it seems unlikely to achieve full successful treatment results.

## CONCLUSION

Although extra-genital condylomas are rare, such large intraoral condylomas are much rarer. In the presence of oral condylomas, the possibility of genital condylomas should be brought to mind and the patient should be re-evaluated from this perspective. Surgical removal of an oral condyloma of this size may be troublesome. Thus, it is important to make the surgical excision of such large condylomas in a tertiary medical center and to take care of some surgical points during the procedure.

## Notes

***Note: ***This case was presented as a poster presentation at the annual meeting of Oral and Maxillofacial Surgery Society in Antalya, Turkey in 2010.

## References

[1] Anderson KM, Perez-Montiel D, Miles L, Allen CM, Nuovo GJ (2003). The histologic differentiation of oral condylomaacuminatum from its mimics. Oral Surg Oral Med Oral Pathol Oral Radiol Endod.

[2] Tan XJ, Wu M, Lang JH (2010). Giant condylomaacuminatum of the vulva. Int J Infect Dis.

[3] Rimkevicius A, Puriene A, Gaigalas M (2011). Condylomaacuminatum: some aspects. Acta Medica Lituanica.

[4] Dos Reis HL, Rabelo PC, Santana MR, Ferreira DC, Filho AC (2009). Oral squamous papilloma and condylomaacuminatum as manifestations of buccal-genital infection by human papillomavirus. Ind J Sex Transm Dis.

[5] Shafer WG, Hine MK, Levy BM (1974). A textbook of oral pathology.

[6] American College of Obstetricians and Gynecologists ACOG Practice Bulletin. Clinical Management Guidelines for Obstetrician-Gynecologists. Number 61, April 2005. Human Papillomavirus. Obstet Gynecol.

[7] Workowski KA, Berman SM (2002). CDC sexually transmitted diseases treatment guidelines. Clin Infect Dis.

[8] Weinstock H, Berman S, Cates W Jr (2004). Sexually transmitted diseases among American youth: incidence and prevalence estimates, 2000. Perspect Sex Reprod Health.

[9] Doorbar J (2005). The papillomavirus life cycle. J ClinVirol.

[10] Gupta RR, Puri UP, Mahajan BB, Sahni SS, Garg G (2001). Intraoral giant condylomaaccuminatum. Indian J Dermatol Venerol Leprol.

